# ML281 Does Not Function as an STK33 Inhibitor but Modulates Proliferation and Differentiation of Keratinocytes Derived from Hypertrophic Scars

**DOI:** 10.3390/ijms27188273

**Published:** 2026-09-17

**Authors:** Ya Xin Zheng, Hui Song Cui, You Ra Lee, So Young Joo, Yoon Soo Cho, In Suk Kwak, Cheong Hoon Seo

**Affiliations:** 1 Burn Institute, Department of Rehabilitation Medicine, College of Medicine, Hangang Sacred Heart Hospital, Hallym University, 94-200 Yeongdeungpo-Dong, Yeongdeungpo-Ku, Seoul 07247, Republic of Korea; 2 Department of Rehabilitation Medicine, College of Medicine, Hangang Sacred Heart Hospital, Hallym University, 94-200 Yeongdeungpo-Dong, Yeongdeungpo-Ku, Seoul 07247, Republic of Korea; 3 Department of Rehabilitation Medicine, National Fire Hospital, Chungbuk Innovation City 27740, Republic of Korea; 4 Seoul National University Hospital, Seoul 03080, Republic of Korea; 5 Department of Anesthesiology and Pain Medicine, Hangang Sacred Heart Hospital, College of Medicine, Hallym University, Seoul 07247, Republic of Korea

**Keywords:** STK33, ML281, post-burn hypertrophic scar, keratinocytes

## Abstract

Post-burn hypertrophic scar (HTS) formation is influenced by the dynamic balance between keratinocyte proliferation and differentiation, a process critical for maintaining skin homeostasis. Serine/threonine kinase 33 (STK33) has emerged as a potential therapeutic target in oncology. However, its role in HTS formation remains unclear, and its effects on keratinocytes are poorly understood. We isolated human HTS-derived keratinocytes (HTSKs) from post-burn HTS tissues and treated them with ML281, originally developed as an STK33 inhibitor. We examined markers associated with keratinocyte phenotypes and functions, including proliferation (proliferating cell nuclear antigen, c-Myc, keratins 5, 14, 6, 16, and 17), epithelial–mesenchymal transition (EMT; snail1, slug, twist1, e-cadherin, n-cadherin, and vimentin), differentiation (keratins 1 and 10, involucrin, loricrin, Notch1, p21, and p27), and apoptosis (cytochrome c, cleaved caspase3, Bid, Bad, Bax, and Bcl-2). mRNA and protein expression levels were assessed using reverse transcription–quantitative PCR, Western blotting, and immunocytochemistry. In HTSKs, ML281 increased STK33 enzymatic activity and STK33 mRNA and protein expression, rather than inhibiting STK33 activity. ML281 treatment reduced the expression of proliferation-associated markers, promoted an EMT-associated phenotype, modulated the expression of differentiation-associated markers, and induced apoptosis-associated changes. Collectively, these findings suggest that ML281 alters post-burn HTSK phenotypes associated with proliferation, EMT, differentiation, and apoptosis.

## 1. Introduction

Burn injuries, predominantly caused by flames and scalding, are associated with substantial morbidity and mortality. Severe burns can induce systemic pathophysiological changes that affect nearly every organ system [1]. Early management of burn wounds has traditionally focused on preventing infection and alleviating pain [2]. Subsequently, with the substantial reduction in burn-related mortality, therapeutic objectives for post-burn wound healing have progressively shifted toward managing long-term burn sequelae [2]. Burn injuries frequently result in a range of sensory disturbances, including pain, pruritus, and hypoesthesia. These persistent symptoms can significantly compromise quality of life, contributing to sleep disturbances and limitations in daily activities and thereby imposing a substantial psychosocial burden [3]. However, complete scarless wound healing has not yet been achieved in clinical practice. Pathological fibrosis occurs in up to 70% of wounds, predominantly manifesting as keloids and hypertrophic scars (HTS) [4]. Despite advances in research, surgical excision remains the gold standard for the treatment of HTS. Perioperatively, nonsurgical modalities, including massage, laser therapy, extracorporeal shockwave therapy, and pharmacological agents, are frequently used to limit scar development and optimize scar appearance and function [5].

In this study, we focused on keratinocytes, the predominant cell type in the epidermis. In normal skin, keratinocytes constitute a self-renewing population. The basal layer contains proliferating keratinocytes that initiate a differentiation program as they migrate upward, becoming progressively flatter and sequentially forming the spinous and granular layers. Ultimately, terminally differentiated keratinocytes in the stratum corneum lose their organelles and are exfoliated as dead cells [6,7]. The dynamic balance between keratinocyte proliferation and differentiation is critical for maintaining epidermal homeostasis [8]. Under normal physiological conditions, approximately 85% of keratinocytes in the basal layer are quiescent [7]. During wound healing, the population of migratory keratinocytes peaks in the inflammatory phase. Concurrently, keratinocytes originating from both the basal and suprabasal layers undergo partial epithelial–mesenchymal transition (EMT), accompanied by reduced intercellular adhesion and migration toward the wound site [9,10]. During the proliferative phase, the population of proliferating keratinocytes increases, and these cells differentiate to form a continuous monolayer, thereby completing re-epithelialization, a hallmark and fundamental requirement of wound closure that enables the skin to restore its barrier function [9,11].

HTS formation, driven by aberrant wound healing, is characterized by a marked imbalance between keratinocyte proliferation and differentiation [8]. Using an in vitro monolayer culture system, we previously demonstrated that exosomes derived from HTS fibroblasts concurrently induce hyperactivation, hyperproliferation, and aberrant differentiation in normal human keratinocytes [12]. Post-burn HTS is accompanied by keratinocyte activation and hyperproliferation, with markedly increased epidermal thickness compared with that in normal skin [13]. Excessive keratinocyte proliferation impairs their migration and differentiation, thereby compromising re-epithelialization efficiency and delaying wound closure. In vitro studies have demonstrated that hyperactivated keratinocytes exhibit reduced lateral migratory capacity [14]. Chronic wound formation is characterized by keratinocyte hyperproliferation, ultimately leading to impaired terminal differentiation and parakeratosis, characterized by retention of nuclei in the stratum corneum [15,16].

Serine/threonine kinase 33 (STK33) has been investigated as a potential therapeutic target in oncology. Accumulating evidence from both in vivo and in vitro studies indicates that STK33 knockdown by RNA interference (RNAi) modulates the development and progression of multiple cancer types, resulting in reduced proliferation, migration, and invasion and increased apoptosis [17,18]. To date, the involvement of STK33 in wound healing and HTS formation, as well as its specific effects on keratinocytes, has not been investigated. In this study, ML281, originally developed to inhibit STK33, was chosen as the pharmacological tool to explore the potential involvement of STK33 in HTS-derived keratinocytes. ML281 has demonstrated high selectivity for STK33, with only two additional kinases showing marginal inhibition (≥25%) [19]. Previous studies have demonstrated that ML281 suppresses human small-cell lung carcinoma cells by inducing apoptosis [20]. In HTS, one of its characteristics is significantly diminished keratinocyte apoptosis [21]. Based on these findings, we hypothesized that ML281 may modulate the pathological phenotype of human HTS-derived keratinocytes (HTSKs). In this study, we assessed a panel of markers associated with proliferation, EMT, differentiation, and apoptosis in HTSKs to determine whether ML281 treatment affects their pathological phenotype and to explore its potential relevance to HTS formation.

## 2. Results

### 2.1. Effects of ML281 Treatment on STK33 Activity in HTSKs

**Figure 1:**
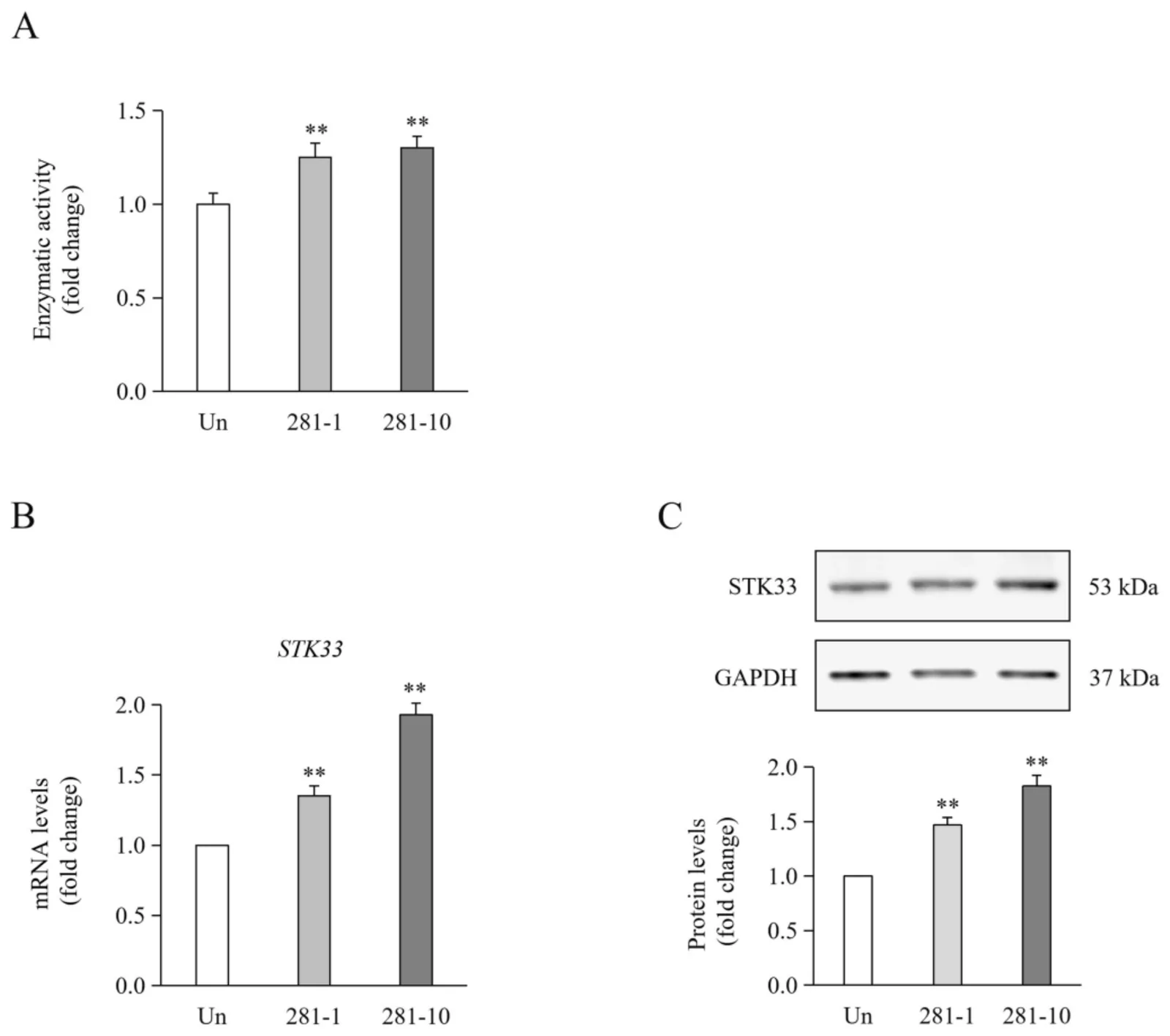
ML281 treatment modulated the enzymatic activity and expression of STK33 in HTSKs. Treatment with ML281 for 48 h significantly induced enzymatic activity (**A**) and protein expression (**C**), but had no significant effect on mRNA expression (**B**) of STK33 (*STK33*). Fold changes were normalized to 1.0 in untreated HTSKs. Each group comprised four biological replicates (n = 4). ** *p* < 0.01, vs. the untreated group. Un, untreated HTSKs; 1 and 10, ML281 concentrations (μM).

In KRAS-dependent cancer cell lines, 1 μM ML281 has been reported to inhibit STK33 activity by approximately 60% [19]. However, in the present study, STK33 enzymatic activity in HTSKs was significantly increased following treatment with 1 and 10 μM ML281 (*p* < 0.01; Figure 1A), accompanied by significant increases in both STK33 mRNA and protein expression levels (*p* < 0.01; Figure 1A,C). Taken together, these findings suggest that ML281 does not suppress STK33 activity in HTSKs.

### 2.2. Effects of ML281 Treatment on Proliferation-Associated Markers in HTSKs

Keratins are major structural proteins produced by keratinocytes that form the intermediate filament cytoskeleton through heterodimerization of type I and type II keratin pairs [22]. In the normal epidermis, proliferating keratinocytes in the basal layer are characterized by high expression of keratin 14 (KRT14) and 5 (KRT5) [22]. Under pathological conditions, keratins 6 (KRT6), 16 (KRT16), and 17 (KRT17) are prominently expressed and replace KRT14 and KRT5 during hyperproliferation in the basal layer [22]. Additionally, proliferating cell nuclear antigen (PCNA) and c-Myc are well-recognized as markers associated with cell proliferation [16].

**Figure 2:**
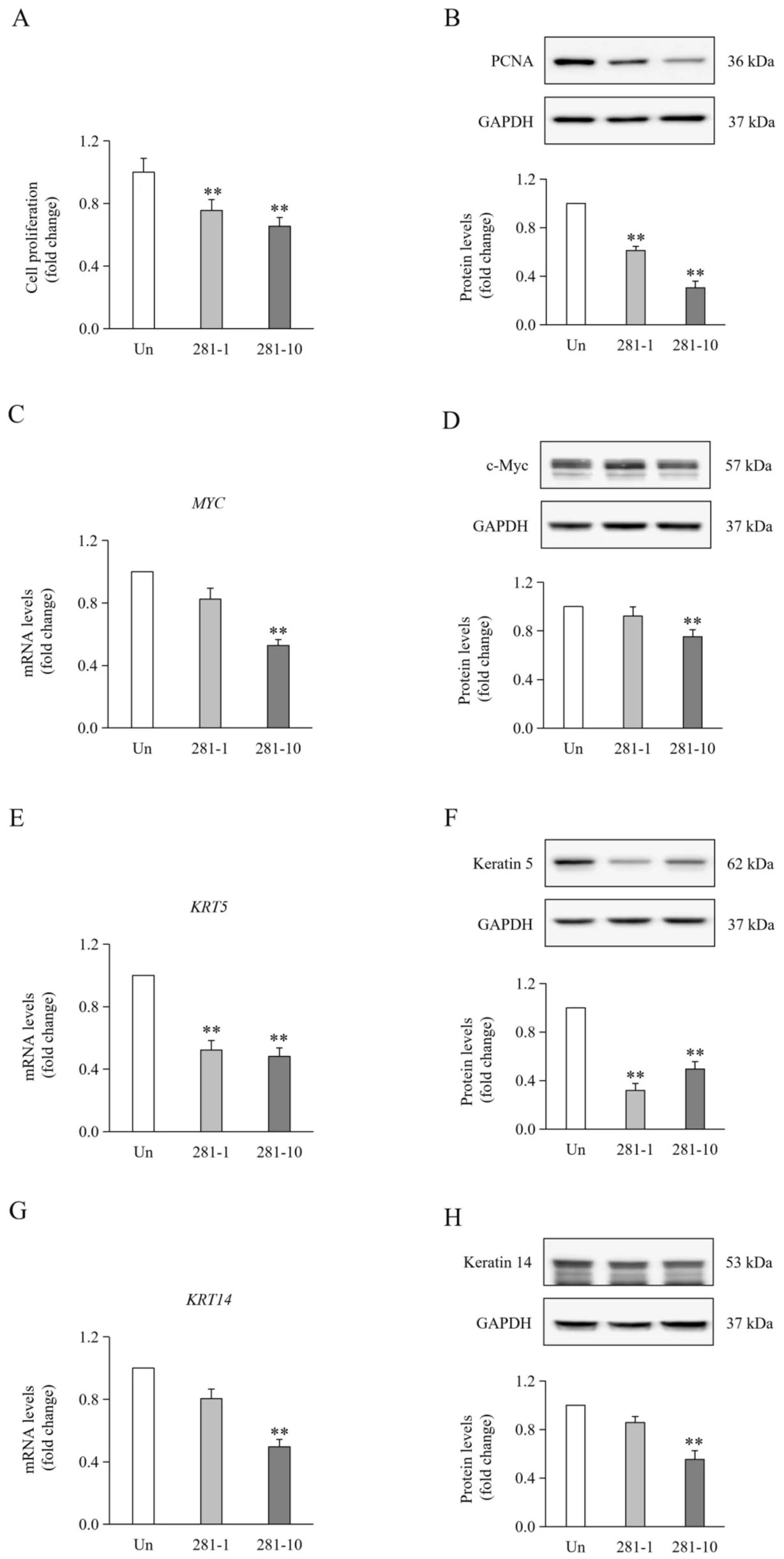
ML281 treatment modulated the expression of proliferation-associated markers in HTSKs. Treatment with ML281 for 48 h significantly reduced cell proliferation (**A**), mRNA and protein expression of PCNA (**B**), c-Myc (*MYC*) (**C**,**D**), keratin 5 (*KRT5*) (**E**,**F**), and keratin 14 (*KRT14*) (**G**,**H**). Fold changes were normalized to 1.0 in untreated HTSKs. Each group comprised four biological replicates (n = 4). ** *p* < 0.01, vs. the untreated group. Un, untreated HTSKs; 1 and 10, ML281 concentrations (μM).

**Figure 3:**
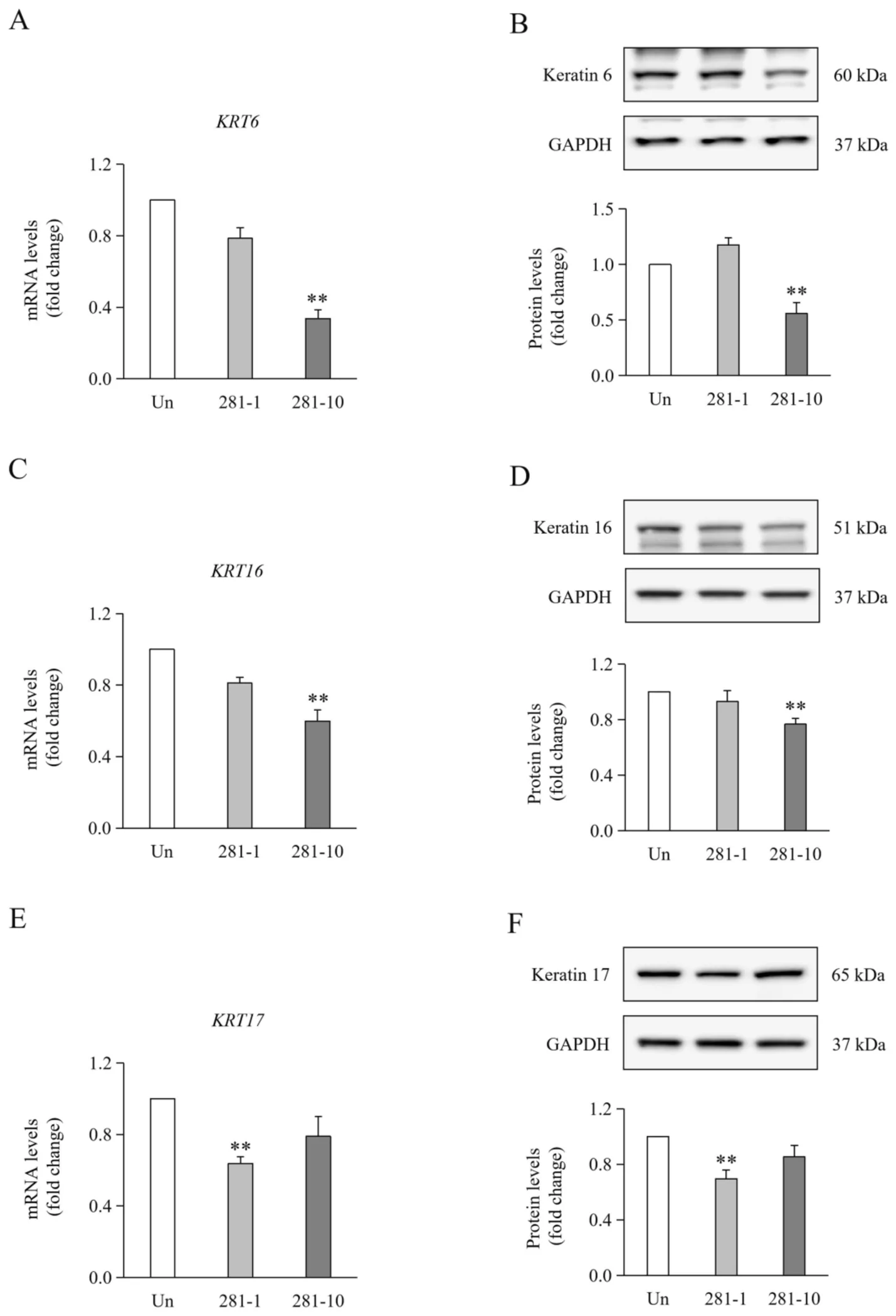
ML281 treatment modulated the expression of proliferation-associated markers in HTSKs. Treatment with ML281 for 48 h significantly reduced mRNA and protein expression of keratin 6 (KRT6) (**A**,**B**), keratin 16 (KRT16) (**C**,**D**), and keratin 17 (KRT17) (**E**,**F**). Fold changes were normalized to 1.0 in untreated HTSKs. Each group comprised four biological replicates (n = 4). ** *p* < 0.01, vs. the untreated group. Un, untreated HTSKs; 1 and 10, ML281 concentrations (μM).

**Figure 4:**
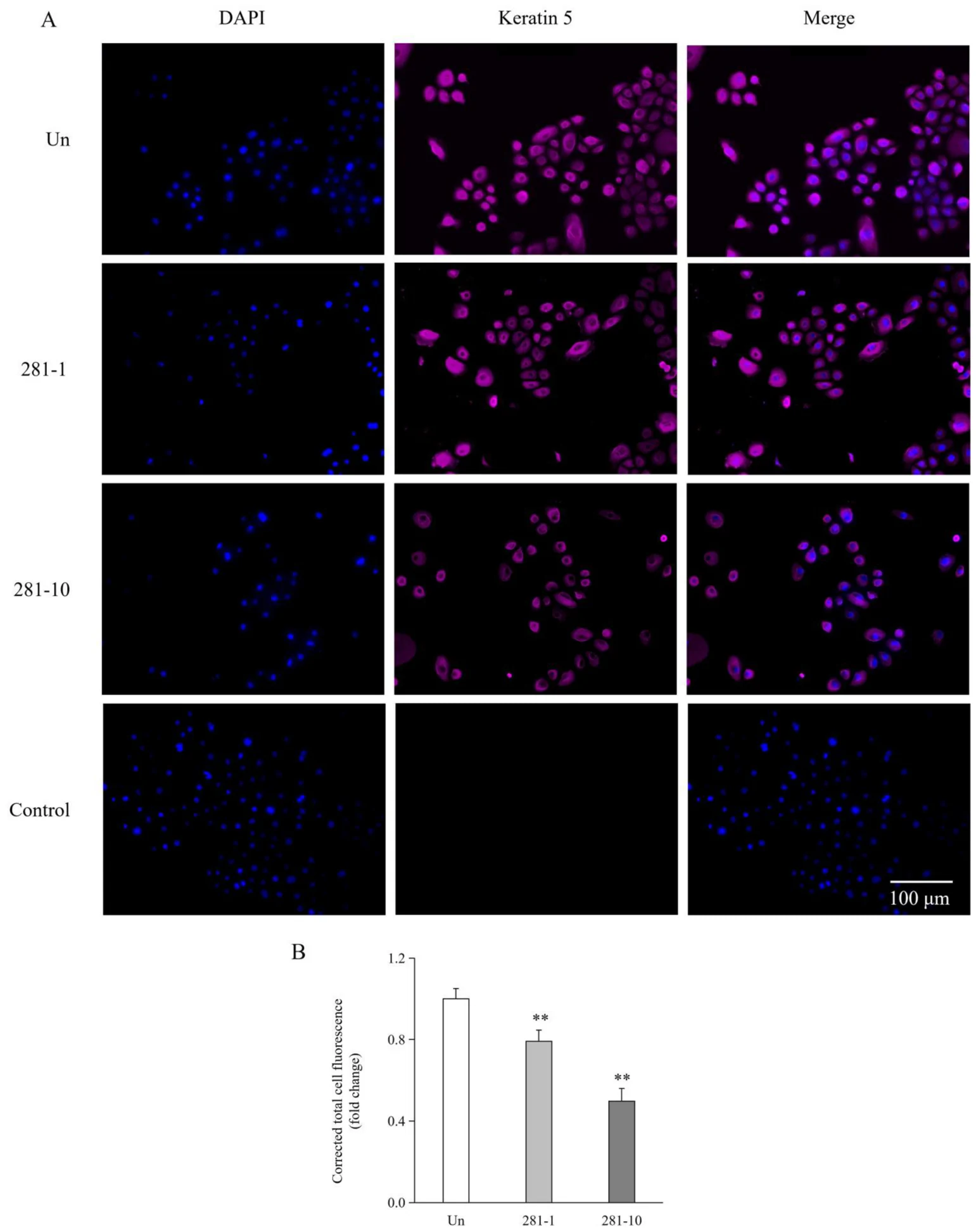
ML281 treatment modulated the expression of proliferation-associated markers in HTSKs. Representative images are shown for DAPI staining, ICC staining, merged images, and isotype control respectively (**A**). Scale bar = 100 μm. Treatment with ML281 for 48 h significantly reduced immunofluorescence intensity of keratin 5 (**B**). Fold changes were normalized to 1.0 in untreated HTSKs. Each group comprised four biological replicates (n = 4). ** *p* < 0.01, vs. the untreated group. Un, untreated HTSKs; 1 and 10, ML281 concentrations (μM).

Compared with the control, ML281 significantly suppressed HTSK proliferation in a concentration-dependent manner, with significant inhibition observed at 1 and 10 μM (*p* < 0.01; Figure 2A). ML281 also significantly decreased PCNA protein expression at both concentrations (*p* < 0.01; Figure 2B). At 10 μM, ML281 treatment significantly reduced mRNA and protein levels of c-Myc (*p* < 0.01; Figure 2C,D). In addition, ML281 significantly reduced mRNA and protein levels of KRT5 at 1 and 10 μM (*p* < 0.01; Figure 2E,F). At 10 μM, ML281 treatment also significantly reduced mRNA and protein levels of KRT14 (*p* < 0.01; Figure 2G,H), KRT6 (*p* < 0.01; Figure 3A,B), and KRT16 (*p* < 0.01; Figure 3C,D). However, mRNA and protein levels of KRT17 were significantly reduced only at 1 μM ML281 (*p* < 0.01; Figure 3E,F). The reduction in KRT5 protein expression was further confirmed by immunocytochemistry (ICC; *p* < 0.01; Figure 4).

Collectively, these results indicate that ML281 treatment suppresses proliferation in HTSKs.

### 2.3. Effects of ML281 Treatment on EMT-Associated Markers in HTSKs

EMT is a cellular process associated with keratinocyte migration and involves the upregulation of EMT-associated transcription factors, including twist1, snail1, and slug, accompanied by downregulation of the epithelial marker e-cadherin and upregulation of the mesenchymal markers n-cadherin and vimentin [10,23].

**Figure 5:**
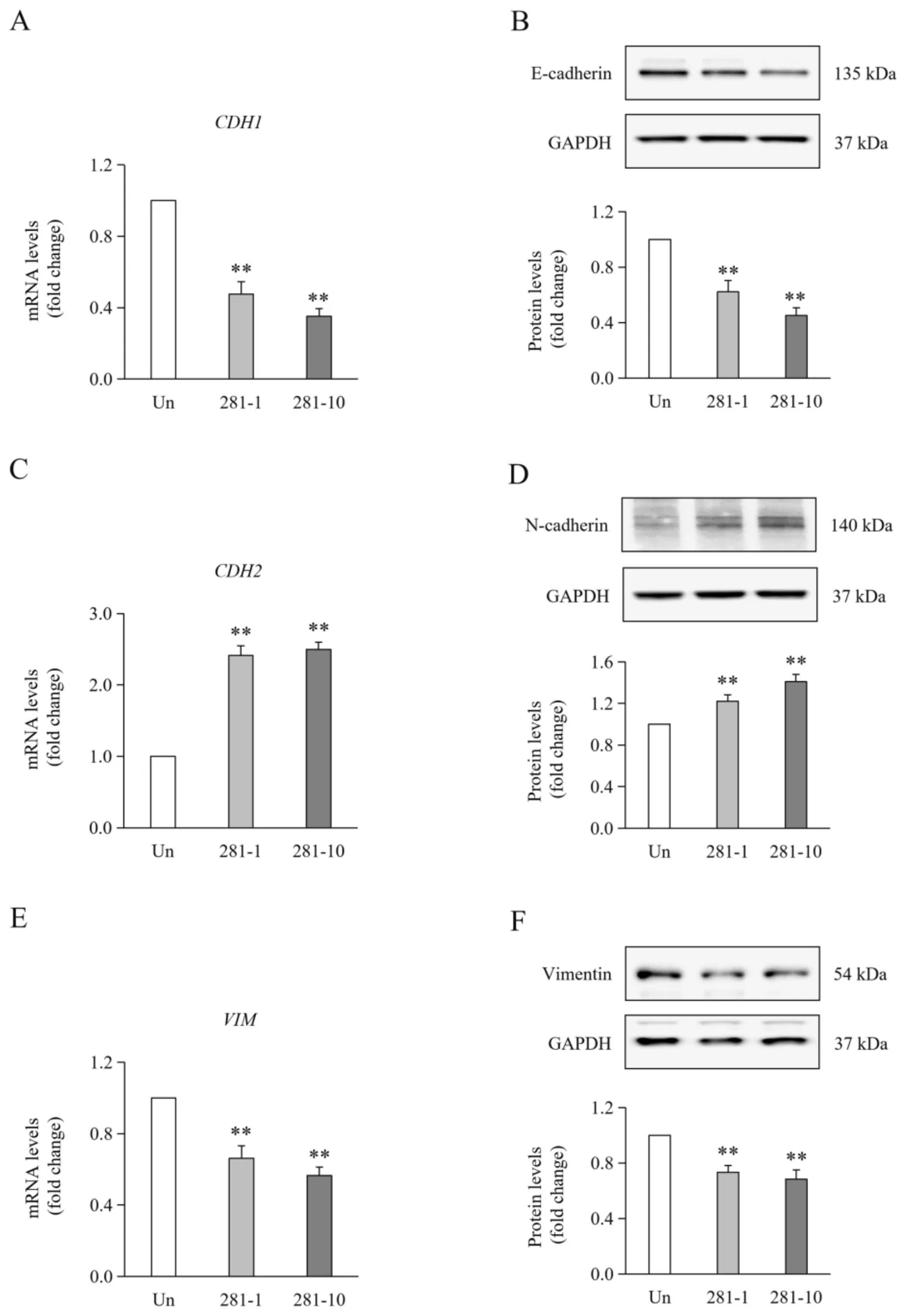
ML281 treatment modulated the expression of EMT-associated markers in HTSKs. Treatment with ML281 for 48 h significantly reduced mRNA and protein expression of E-cadherin (*CDH1*) (**A**,**B**) and vimentin (*VIM*) (**E**,**F**), and induced expression of N-cadherin (*CDH2*) (**C**,**D**). Fold changes were normalized to 1.0 in untreated HTSKs. Each group comprised four biological replicates (n = 4). ** *p* < 0.01, vs. the untreated group. Un, untreated HTSKs; 1 and 10, ML281 concentrations (μM).

**Figure 6:**
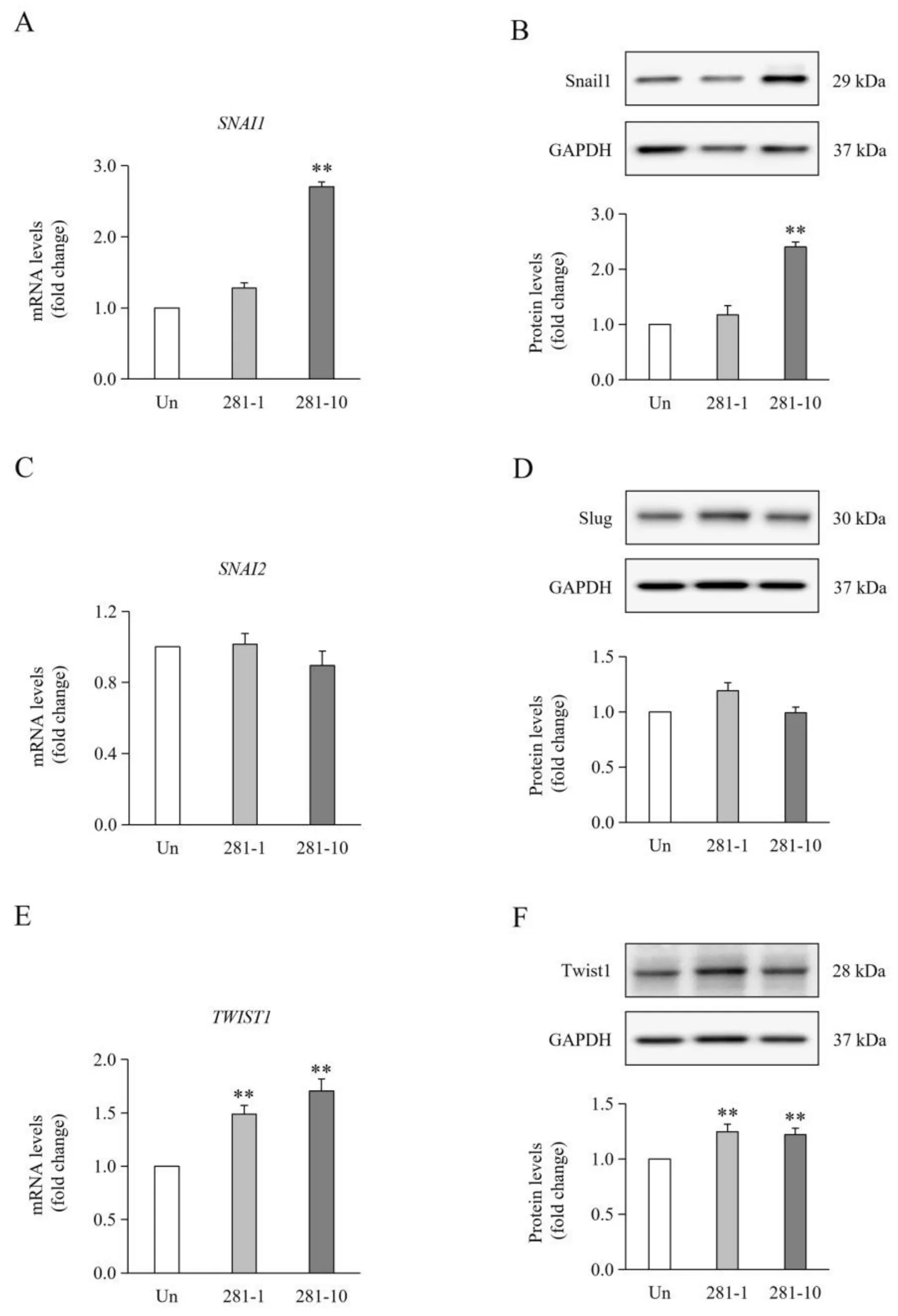
Treatment with ML281 for 48 h significantly induced mRNA and protein expression of snail1 (*SNAI1*) (**A**,**B**) and twist1 (*TWIST1*) (**E**,**F**), but had no significant effect on slug (*SNAI2*) (**C**,**D**). Fold changes were normalized to 1.0 in untreated HTSKs. Each group comprised four biological replicates (n = 4). ** *p* < 0.01, vs. the untreated group. Un, untreated HTSKs; 1 and 10, ML281 concentrations (μM).

Compared with the control, ML281 significantly reduced mRNA and protein levels of e-cadherin at 1 and 10 μM (*p* < 0.01; Figure 5A,B), whereas it significantly increased mRNA and protein levels of n-cadherin (*p* < 0.01; Figure 5C,D) and vimentin (*p* < 0.01; Figure 5E,F) at both concentrations. At 10 μM, ML281 significantly increased snail1 mRNA and protein levels (*p* < 0.01; Figure 6A,B), whereas mRNA and protein levels of slug remained unchanged (*p* > 0.05; Figure 6C,D). Furthermore, ML281 significantly increased mRNA and protein levels of twist1 (*p* < 0.01; Figure 6E,F) at both concentrations. However, ML281 treatment at both 1 and 10 μM significantly suppressed the migration of HTSKs compared with untreated controls (1 μM: *p* < 0.05; 10 μM: *p* < 0.01; Supplementary Figure S1).

Taken together, these findings indicate that ML281 treatment modulates an EMT-associated phenotype and migratory capacity in HTSKs.

### 2.4. Effects of ML281 Treatment on Differentiation-Associated Markers in HTSKs

In the normal epidermis, keratin 1 (KRT1) and 10 (KRT10) are highly expressed in keratinocytes of the spinous layer [22]. Other structural proteins may also serve as markers of keratinocyte differentiation. During the early stages of differentiation, involucrin is synthesized as keratinocytes exit the basal layer [24]. During the later stages of terminal differentiation, loricrin is predominantly expressed in the granular layer [25]. Caspase14 is expressed during terminal keratinocyte differentiation, with its expression increasing at the interface between the granular layer and the stratum corneum, where it is regulated by transcription factors activated during terminal keratinocyte differentiation [26].

**Figure 7:**
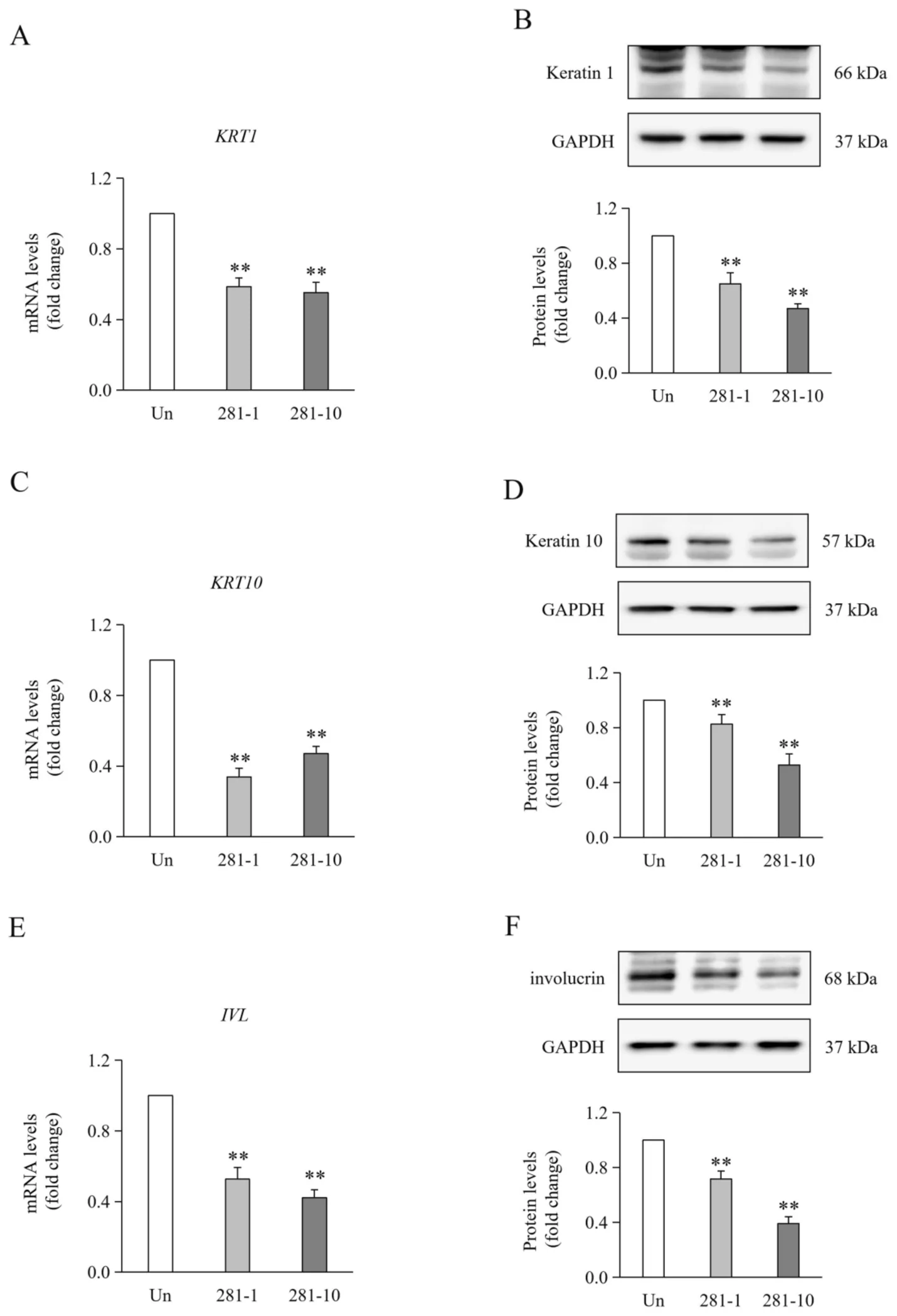
ML281 treatment modulated the expression of differentiation-associated markers in HTSKs. Treatment with ML281 for 48 h significantly reduced the mRNA and protein expression levels of keratin 1 (*KRT1*) (**A**,**B**), keratin 10 (*KRT10*) (**C**,**D**), and involucrin (*IVL*) (**E**,**F**). Expression levels were normalized to 1.0 in untreated HTSKs. Each group comprised four biological replicates (n = 4). ** *p* < 0.01, vs. the untreated group. Un, untreated HTSKs; 1 and 10, ML281 concentrations (μM).

**Figure 8:**
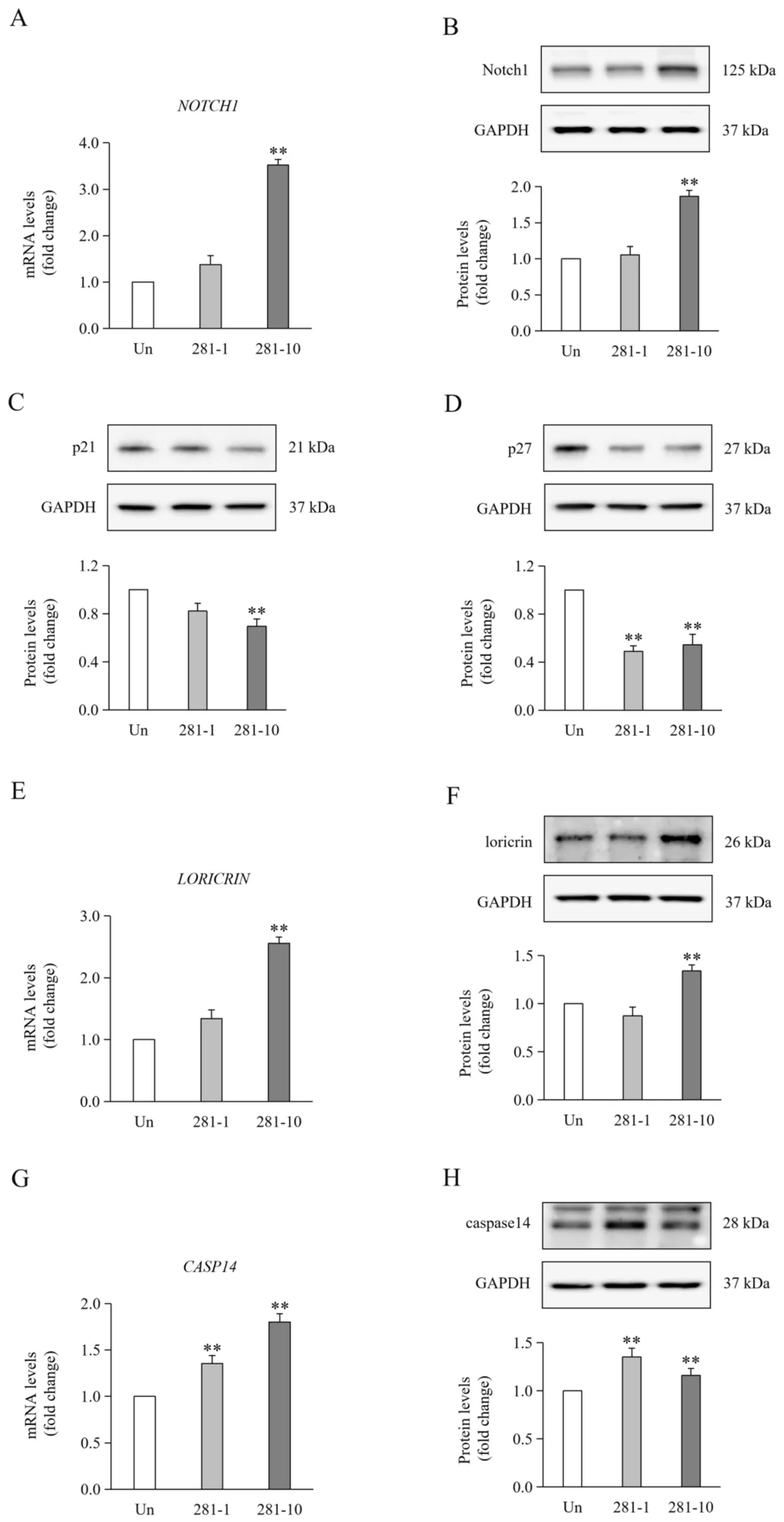
ML281 treatment modulated the expression of differentiation-associated markers in HTSKs. Treatment with ML281 for 48 h significantly induced mRNA and protein expression of Notch1 (*NOTCH1*) (**A**,**B**), loricrin (*LORICRIN*) (**E**,**F**), and caspase14 (*CASP14*) (**G**,**H**), and reduced protein expression of p21 (**C**) and p27 (**D**). Expression levels were normalized to 1.0 in untreated HTSKs. Each group comprised four biological replicates (n = 4). ** *p* < 0.01, vs. the untreated group. Un, untreated HTSKs; 1 and 10, ML281 concentrations (μM).

**Figure 9:**
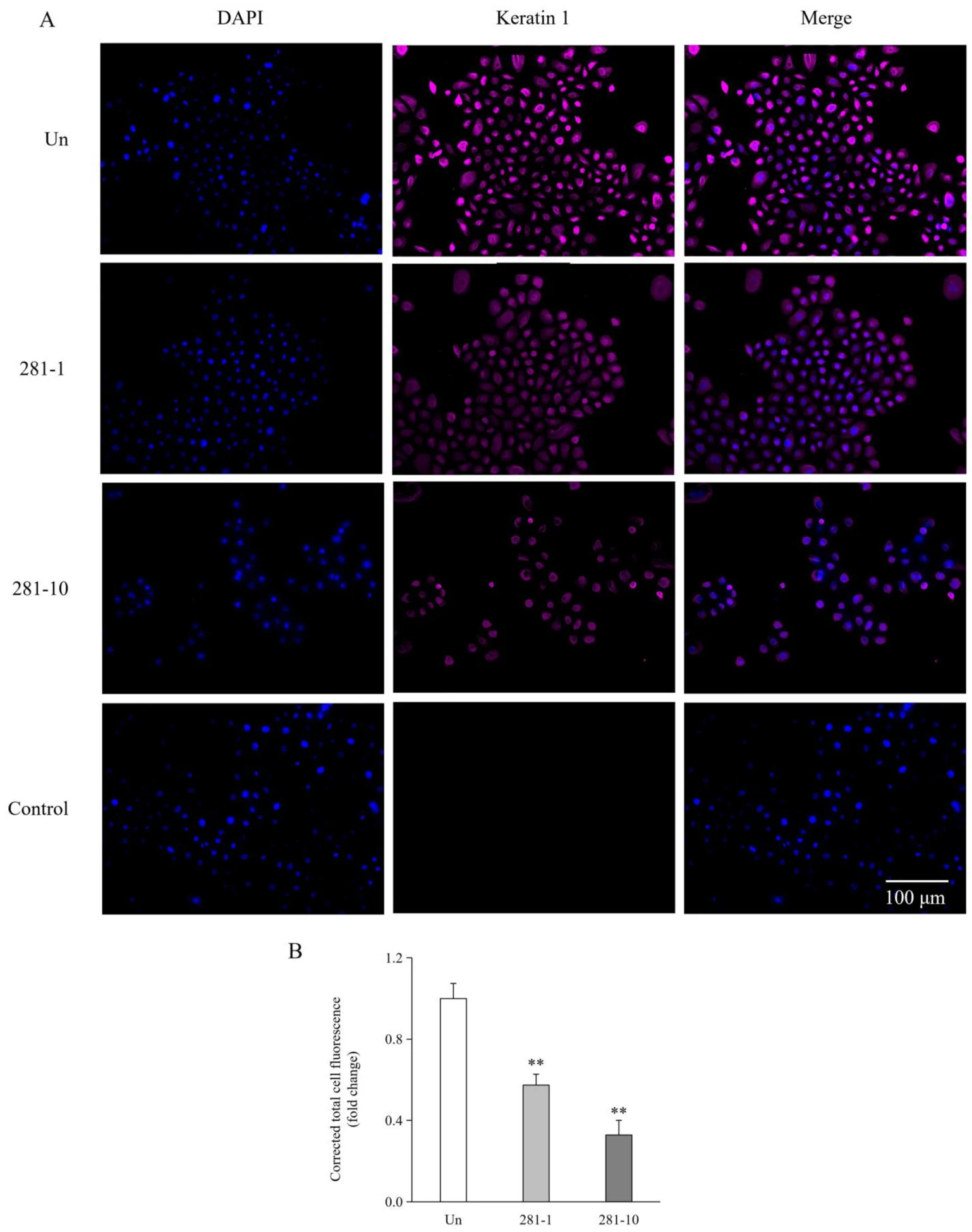
ML281 treatment modulated the expression of differentiation-associated markers in HTSKs. Representative images are shown for DAPI staining, ICC staining, merged images, and isotype control respectively (**A**). Scale bar = 100 μm. Treatment with ML281 for 48 h significantly reduced immunofluorescence intensity of keratin 1 (**B**). Fold changes were normalized to 1.0 in untreated HTSKs. Each group comprised four biological replicates (n = 4). ** *p* < 0.01, vs. the untreated group. Un, untreated HTSKs; 1 and 10, ML281 concentrations (μM).

**Figure 10:**
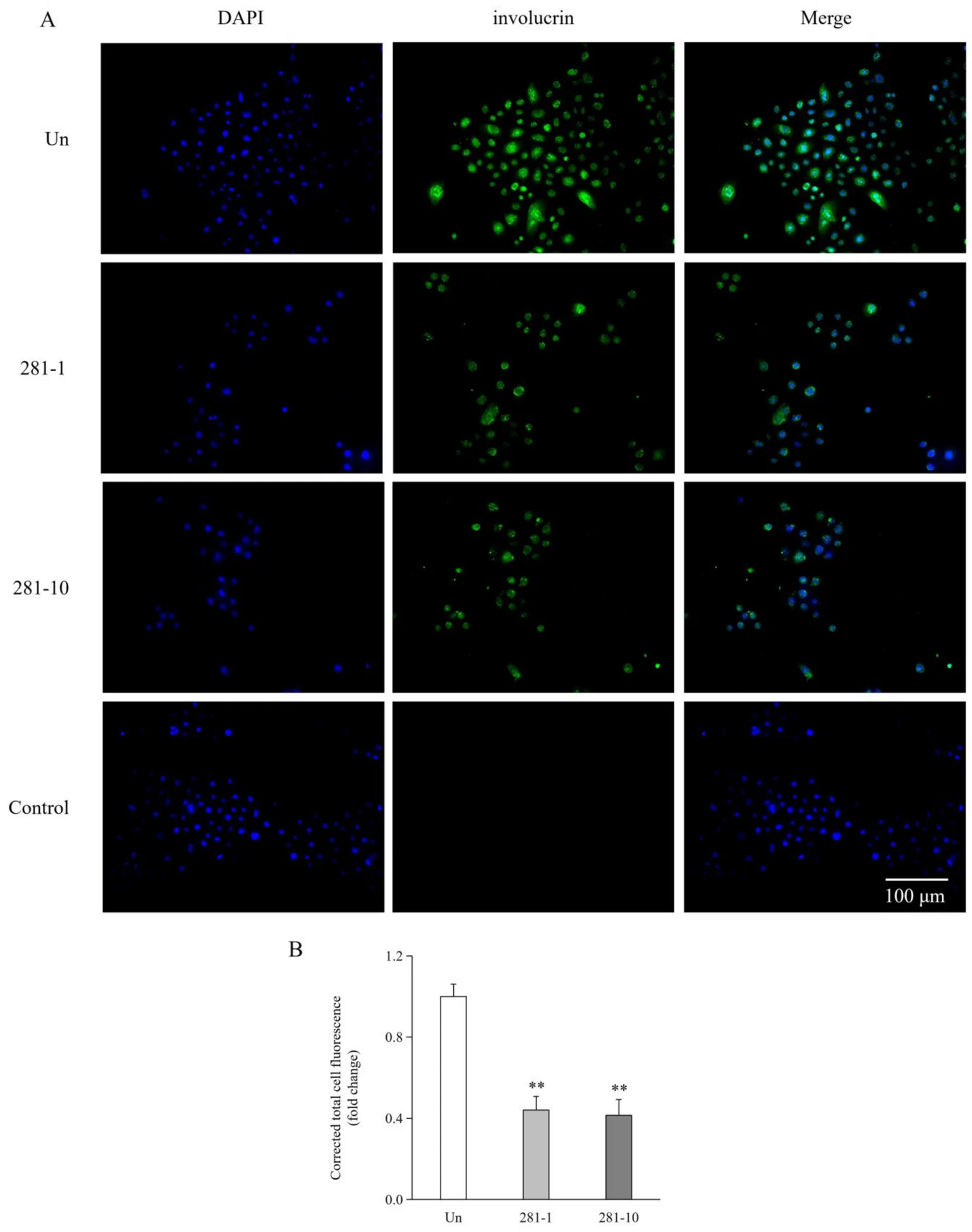
ML281 treatment modulated the expression of differentiation-associated markers in HTSKs. Representative images are shown for DAPI staining, ICC staining, merged images, and isotype control respectively (**A**). Scale bar = 100 μm. Treatment with ML281 for 48 h significantly reduced the immunofluorescence intensity of involucrin (**B**). Fold changes were normalized to 1.0 in untreated HTSKs. Each group comprised four biological replicates (n = 4). ** *p* < 0.01, vs. the untreated group. Un, untreated HTSKs; 1 and 10, ML281 concentrations (μM).

Compared with the control, ML281 significantly reduced mRNA and protein levels of KRT1 (*p* < 0.01; Figure 7A,B), KRT10 (*p* < 0.01; Figure 7C,D), and involucrin (*p* < 0.01; Figure 7E,F) at 1 and 10 μM. At 10 μM, ML281 significantly increased the mRNA and protein levels of Notch1 (*p* < 0.01; Figure 8A,B). In addition, ML281 significantly reduced p21 protein levels at 10 μM (*p* < 0.01; Figure 8C) and p27 protein levels at both concentrations (*p* < 0.01; Figure 8D). ML281 also significantly increased mRNA and protein levels of loricrin at 10 μM (*p* < 0.01; Figure 8E,F) and caspase14 at 1 and 10 μM (*p* < 0.01; Figure 8G,H). Consistent with these results, ICC analysis revealed reduced KRT1 (*p* < 0.01; Figure 9) and involucrin (*p* < 0.01; Figure 10) expression at both ML281 concentrations.

Collectively, these results indicate that ML281 treatment modulates the expression of differentiation-associated markers in HTSKs.

### 2.5. Effects of ML281 Treatment on Apoptosis-Associated Changes in HTSKs

During apoptosis, Bid activation promotes mitochondrial depolarization and cytochrome c release, thereby activating caspase3 and triggering apoptosis [21]. The Bcl-2 family plays a critical regulatory role in this process: Bcl-2 preserves mitochondrial membrane integrity and exerts anti-apoptotic effects, whereas Bad and Bax facilitate apoptosis by antagonizing Bcl-2 [21].

**Figure 11:**
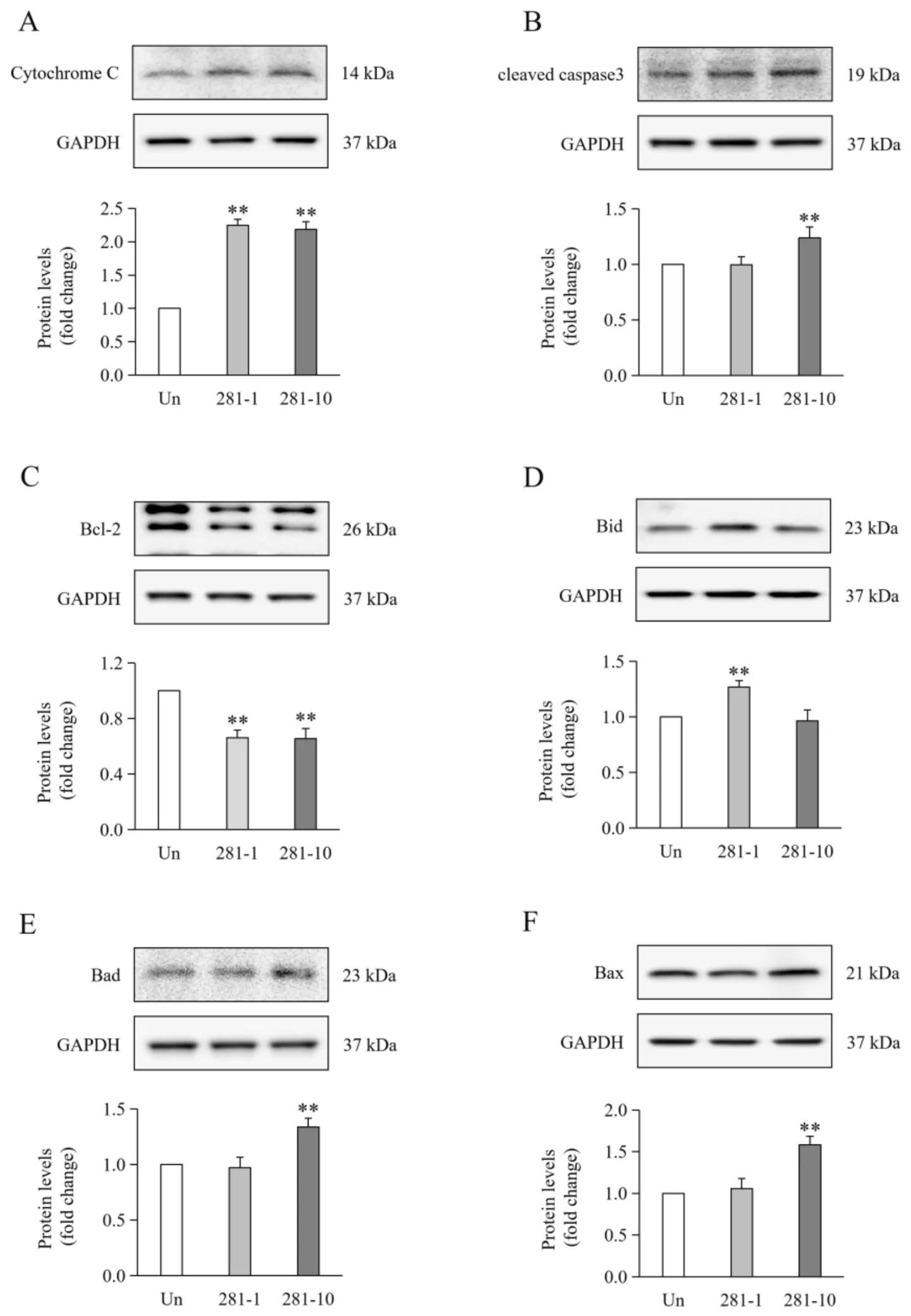
ML281 treatment modulated the expression of apoptosis-associated markers in HTSKs. Treatment with ML281 for 48 h significantly induced protein expression of cytochrome C (**A**), cleaved caspase3 (**B**), Bid (**D**), Bad (**E**), and Bax (**F**), and reduced expression of Bcl-2 (**C**). Expression levels were normalized to 1.0 in untreated HTSKs. Each group comprised four biological replicates (n = 4). ** *p* < 0.01, vs. the untreated group. Un, untreated HTSKs; 1 and 10, ML281 concentrations (μM).

Compared with the control, ML281 significantly increased cytochrome c protein levels at 1 and 10 μM (*p* < 0.01; Figure 11A). At 10 μM, ML281 significantly increased cleaved caspase3 (*p* < 0.01; Figure 11B). ML281 significantly reduced Bcl-2 protein levels at 1 and 10 μM (*p* < 0.01; Figure 11C). At 1 μM, ML281 significantly increased Bid (*p* < 0.01; Figure 11D), whereas at 10 μM, it significantly increased Bad (*p* < 0.01; Figure 11E) and Bax (*p* < 0.01; Figure 11F) protein levels. Taken together, these findings indicate that ML281 treatment promotes apoptosis-associated changes in HTSKs.

## 3. Discussion

First of all, our current findings should not be interpreted as effects of STK33 inhibition, as ML281 did not inhibit STK33 activity in HTSKs; rather, STK33 activity increased following ML281 treatment. We proposed that this response may be attributable to cell type-specific differences in dependence on STK33 modulation. STK33 was originally identified as a selective target in KRAS-dependent cancers [18]. The sensitivity of KRAS-dependent cancer cell lines to STK33 inhibition has been attributed to STK33-dependent adaptive changes acquired during malignant transformation [18]. On the other hand, such paradoxical activation has been previously reported and attributed to mechanisms such as conformational changes in the kinase domain or inhibitor-mediated activation of kinase signaling [27,28].

STK33 has been implicated in the regulation of cell survival and proliferation, particularly in KRAS-dependent cancer cell lines derived from hematopoietic and epithelial tissues [19]. For instance, in hepatocellular carcinoma, STK33 overexpression promotes tumor cell proliferation both in vivo and in vitro [29]. However, shRNA-mediated knockdown of STK33 consistently inhibited cell viability and proliferation in KRAS-dependent cancer cell lines but exerted little to no effect in other cancer cell lines or normal human fibroblasts [18]. Therefore, pharmacological inhibition of STK33 may not fully recapitulate the effects of genetic knockdown [18]. For example, BRD-8899, an inhibitor of STK33 kinase activity, failed to affect STK33-mediated proliferation, migration, or invasion and had no effect on cancer cell viability [17,30]. Moreover, some studies have reported conflicting findings, showing that neither STK33 knockdown, expression of a dominant-negative mutant, nor pharmacological inhibition compromises cell survival [31].

In THP-1 cells, ML281 treatment reduces cell viability by approximately 20% [19]. This modest effect has led to the proposal that the observed cytotoxicity might result from off-target effects rather than specific inhibition of STK33 [19]. In this study, ML281 treatment reduced HTSK proliferation by approximately 30%. In post-burn HTS, KRT16, KRT17, KRT6, KRT14, and KRT5 were significantly elevated, whereas compared with that in normal skin [13]. In our previous study, we found a marked upregulation of KRT17 and KRT6 in HTSKs in vitro, accompanied by enhanced proliferation [32]. ML281 treatment significantly downregulated the expression of the proliferation markers described above in HTSKs. This finding prompted us to further assess the balance between proliferation and differentiation in keratinocytes, as this balance is important for determining their activation status based on protein expression profiles [8].

When keratinocytes enter cell-cycle arrest, PCNA and c-Myc levels decrease [33]. During keratinocyte differentiation, KRT10 and KRT1 suppress cell-cycle progression and activate Notch1 while suppressing c-Myc expression, indicating that KRT10 and KRT1 contribute to the opposing and mutually balanced regulation of proliferation and differentiation [22]. During wound healing, KRT1 and KRT10 are downregulated in keratinocytes adjacent to the wound site [22]. During post-burn HTS formation, KRT10 expression showed no significant change [13]. Concurrently, we observed upregulation of KRT1 and involucrin, and downregulation of KRT10 in HTSKs [32]. We hypothesize that the uncoupled expression of KRT1 and KRT10 represents a dysregulated state resulting from disruption of the delicate balance between proliferation and differentiation in HTS formation.

In HTSKs, the Notch signaling pathway is hyperactivated, and inhibition of Notch signaling in vivo has been shown to alleviate HTS formation [34]. Throughout this process, p21 and p27 progressively accumulate until cell-cycle arrest is triggered, facilitating entry into the initial stage of keratinocyte differentiation [35,36]. Knockdown of either p21 or p27 significantly enhances keratinocyte proliferation and markedly downregulates differentiation markers, including KRT1, involucrin, and loricrin [37]. In contrast, during the later stages of keratinocyte differentiation, p21 overexpression has been shown to inhibit terminal differentiation, resulting in reduced p21 expression [38,39]. In a mouse model of keratinocyte carcinogenesis, p21 knockdown enhanced squamous cell differentiation [38]. In differentiated keratinocytes with Notch1 knockdown, p21 expression was significantly reduced, whereas loricrin expression was increased; however, early differentiation markers, including KRT1 and involucrin, remained unchanged [40]. In HTSKs, it showed upregulation of both p21 and p27, suggesting that HTSKs initiate a differentiation program but fail to progress to terminal differentiation, thereby sustaining an activated, hyperproliferative state [32].

In this study, ML281 treatment of HTSKs decreased the expression of early differentiation markers, accompanied by reduced levels of p21 and p27 and increased levels of terminal differentiation markers. These findings indicate that ML281 shifts HTSKs toward a less proliferative state while promoting advanced differentiation, which may ultimately culminate in apoptosis. Regulators of keratinocyte differentiation also regulate apoptosis. For example, Notch signaling has been shown to upregulate the expression of caspase3 during epidermal differentiation [41]. In HaCaT keratinocytes, destabilization and downregulation of p21 protein have been shown to activate caspase3 and caspase7 [42]. Under normal conditions, proliferating keratinocytes in the basal layer are protected from apoptosis by Bcl-2 expression, whereas differentiated keratinocytes in the suprabasal layer lack Bcl-2 and ultimately undergo apoptosis [21]. In HTS, keratinocytes have been previously attributed to the sustained activation of survival signals and anti-apoptotic factors [21]. In HTSKs, Bcl-2 is significantly upregulated compared with that in normal keratinocytes [32]. In this study, ML281 treatment of HTSKs resulted in the downregulation of Bcl-2 and the upregulation of pro-apoptotic markers, suggesting an apoptosis-associated response.

In cancer studies, STK33 has been implicated in the regulation of apoptosis, with its overexpression suppressing apoptosis and its knockdown promoting it [43]. For instance, STK33 knockdown in pancreatic neuroendocrine tumors, renal cell carcinoma, and hypopharyngeal squamous cell carcinoma promotes apoptosis, accompanied by upregulation of caspase3 and cytochrome c and downregulation of Bcl-2 [43,44,45]. In non-cancerous models, reduced STK33 expression has been shown to increase Bax and cleaved caspase3, with flow cytometry and TUNEL assays confirming enhanced apoptosis [46]. Moreover, STK33 has been reported to phosphorylate and inactivate Bad via S6K1 in a kinase activity-dependent manner, thereby inhibiting mitochondria-mediated apoptosis [18]. In human small cell lung carcinoma, ML281 has been demonstrated to promote apoptosis through the S6K1-BAD axis, resulting in increased expression of cleaved caspase9 [20]. However, this effect has been demonstrated only in certain KRAS-dependent cancer cell lines, whereas no effect was observed in U937 and OCI-AML3 cells [18].

Previous studies on STK33 and cell proliferation have often also examined cell migration and invasion. In renal cell carcinoma and hypopharyngeal squamous cell carcinoma, RNAi-mediated knockdown of STK33 has been shown to impair cell proliferation, migration, and invasion, concomitant with e-cadherin upregulation and vimentin downregulation [44,45]. In large-cell lung carcinoma and gastric cancer cell lines, STK33 overexpression increases the expression of EMT-related markers, including twist, snail, slug, vimentin, and n-cadherin, while downregulating β-catenin and e-cadherin, thereby promoting tumor growth and invasion in vivo [17,47]. However, BRD-8899, a small-molecule inhibitor of STK33 kinase activity, has no significant effect on EMT [17].

In this study, ML281 treatment increased the expression of EMT-associated markers, but vimentin expression was reduced and slug expression remained unchanged. In wound healing, keratinocytes exhibit a partial EMT phenotype, and slug has been identified as a critical transcriptional regulator of this process [10]. In hepatocellular carcinoma cells, partial EMT induced by slug overexpression was characterized by downregulation of vimentin [48]. Vimentin and slug have been shown to reciprocally regulate one another. In tumor cells, vimentin serves as a scaffold for the recruitment of slug to ERK, thereby promoting slug phosphorylation, which subsequently enhances vimentin transcription and synergistically drives EMT [49]. In vimentin-deficient mice, wound healing is accompanied by slug suppression and suppression of EMT [50]. Therefore, we hypothesize that partial EMT led to a decrease in vimentin expression, which subsequently led to the lack of slug induction via a feedback loop. However, this hypothesis is preliminary and requires further experimental verification of the vimentin-slug regulatory axis.

During the EMT process, keratinocytes acquired a mesenchymal phenotype that showed characteristic spindle morphology and individual migration [51]. In premalignant human keratinocytes, EMT was accompanied by acquisition of a highly motile and scattered phenotype [52]. However, in our study, cells migrated as keratinocyte colonies, which is associated with cell proliferation, as previously reported [53]. In this process, vimentin expression is predominantly localized to the leading edges of migrating and proliferating colonies, suggesting that this movement is driven by vimentin-containing basal keratinocytes [53]. Vimentin is a key cytoskeletal protein, and its knockdown has been shown to be required for keratinocyte EMT activation, keratinization, and re-epithelialization during wound healing [50]. Vimentin also plays a critical role in cell growth and proliferation and has been reported to promote the proliferation of adjacent cells through TGF-β signaling [54]. In fibroblasts, loss of vimentin has been associated with reduced protein synthesis during wound healing [54]. In vimentin knockdown wounds, certain regions of keratinocyte colonies in the epidermis were poorly organized, imply severely compromise of epidermal migration, maturation, and stratification [50,51]. In addition, enhanced migration is not an obligatory consequence of EMT. In epithelial tumor cells, previous studies have shown that post-EMT cells can exhibit even lower migratory capacity than their pre-EMT counterparts [55]. Accordingly, we hypothesize that reduced vimentin expression observed in this study may cause the reduced migration of keratinocyte colonies.

Taken together, our experiments indicated that ML281 inhibited HTSKs’ proliferation and migration while promoting differentiation and apoptosis. These observations are all in contrast to the functions of upregulated STK33 reported in previous studies. Two main hypotheses may account for these results: one involves off-target effects of ML281 on HTSKs, and the other involves cell type-specific effects of the upregulated STK33 caused by ML281. Previous studies have shown that fibroblasts and keratinocytes can exhibit distinct, and even opposing responses to the same pharmacological agent. For instance, inhibition of the P2X7 receptor promotes the growth and migration of primary keratinocytes but exerts no effect on fibroblasts [56]. In vitro, syringin treatment increased SMAD3 phosphorylation in fibroblasts but produced a nonsignificant decrease in HaCaT cells [57]. Polydeoxyribonucleotide suppresses extracellular signal-regulated kinase (ERK) phosphorylation and collagen accumulation in keratinocytes but promotes both in fibroblasts [58].

Several limitations of this study should be acknowledged. First, HTSKs displayed an unexpected response to ML281 with respect to STK33 activity. The precise mechanism responsible for this paradoxical activation remains unclear. Second, the detailed molecular mechanisms underlying the effects of ML281, specifically its regulation of downstream signaling pathways, were not explored. Third, our findings are based solely on in vitro experiments using primary HTSKs maintained in monolayer culture. Future mechanistic and in vivo studies are required to evaluate the potential therapeutic relevance of ML281 to post-burn HTS.

## 4. Materials and Methods

### 4.1. Keratinocyte Isolation and Culture

The study was conducted in accordance with the principles of the Declaration of Helsinki. Written informed consent was obtained from all patients for the voluntary donation and research use of tissue samples collected during surgery. The demographic and clinical characteristics of the patients included in the study are summarized in Table 1. Fresh tissues were washed with cold Dulbecco’s phosphate-buffered saline (Biowest, Riverside, MO, USA) and cut into approximately 1-mm^3^ pieces for further processing. The tissues were incubated in Dispase II solution (1 unit/mL; Thermo Fisher Scientific, Waltham, MA, USA) at 4 °C for at least 18 h to separate the epidermis from the dermis. The epidermal layer was collected and incubated in type IV collagenase solution (Thermo Fisher Scientific) at 37 °C for 1 h to isolate keratinocytes.

The isolated primary keratinocytes were seeded in T75 flasks (Eppendorf, Hamburg, Germany) and cultured in keratinocyte basal medium (PromoCell GmbH, Heidelberg, Germany) supplemented with 1% antibiotic–antimycotic solution (Thermo Fisher Scientific) and 50 μg/mL gentamicin (Thermo Fisher Scientific). All cultures were maintained at 37 °C in a humidified atmosphere containing 5% CO_2_. When keratinocytes reached 70% confluence, they were treated with 1 or 10 µM ML281 (Selleckchem, Houston, TX, USA), with dimethyl sulfoxide used as the solvent. Cells were harvested using Cello solution (WELGENE, Daegu, Republic of Korea) 24 or 48 h after treatment for RNA and protein analyses, respectively.

### 4.2. STK33 Activity Assay

All subsequent procedures were performed according to the manufacturer’s instructions (V4086 and V6930; Abcam, Cambridge, UK). Keratinocytes were lysed in kinase buffer containing 40 mM Tris-HCl, 20 mM MgCl_2_, 50 μM DL-dithiothreitol, 0.1 mM sodium orthovanadate, and 5 mM β-glycerophosphate disodium. Protein concentrations were determined using a bicinchoninic acid (BCA) protein assay kit (Thermo Fisher Scientific). The kinase reaction mixture was incubated at room temperature for 60 min in a white 96-well plate. Each well contained 20 µL of reaction mixture, comprising 10 µL of cell lysates normalized to an equal protein concentration and 10 µL of substrate/ATP mixture. Next, 20 µL of ADP-Glo™ Reagent was added to each well and incubated for 40 min to deplete the remaining ATP. Finally, Kinase Detection Reagent was added, and the samples were incubated for 30 min before luminescence was measured using a GloMax^®^ Microplate Reader (Promega). A standard curve based on the percentage conversion of ATP to ADP was used to calculate STK33 activity in the samples.

### 4.3. Cell Proliferation Assay

Keratinocytes were seeded in 96-well plates at a density of 1 × 10^5^ cells per well. After reaching 70% confluence, the cells were treated with 1 µM or 10 µM ML281 for 48 h. To evaluate cell viability, an EZ-Cytox cell viability, proliferation, and cytotoxicity assay kit was used (Dogen Bio, Seoul, Republic of Korea). Specifically, 10 μL of the kit reagent was added to 100 μL of culture medium in each well, and the plate was incubated at 37 °C for 2 h. Absorbance was measured at 450 nm and 600 nm using a DTX 880 Multimode Detector (Beckman Coulter, Fullerton, CA, USA). Finally, cell viability was calculated using the following formula:

$$\mathrm{Cell}\;\mathrm{viability}\;(\%)\;=\;\frac{\mathrm{sample}\;\mathrm{absorbance}\;-\;\mathrm{blank}\;\mathrm{absorbance}}{\mathrm{control}\;\mathrm{absorbance}\;-\;\mathrm{blank}\;\mathrm{absorbance}}\times 100$$

### 4.4. ICC Staining

Keratinocytes were seeded onto coated 30 mm coverslips placed in 35 mm culture dishes. Following 48 h of ML-281 treatment, the cells were fixed with 4% paraformaldehyde, permeabilized with PBS containing 0.25% Triton X-100 (PBST; Sigma-Aldrich, St. Louis, MO, USA), and blocked with 10% donkey serum (Sigma-Aldrich). The cells were subsequently incubated overnight at 4 °C with the following primary antibodies: KRT1 (1:200; Abcam; ab93652); KRT5 (1:200; Abcam; ab52635), and involucrin (1:100; Santa Cruz Biotechnology Abcam, Cambridge, UKSanta Cruz Biotechnology, Dallas, TX, USA; sc-21748). To assess non-specific binding, additional untreated control groups were incubated with rabbit monoclonal IgG isotype control (Abcam, ab125938) at the same concentration as the respective primary antibodies. The cells were then washed with PBST and incubated at room temperature in the dark with the appropriate Alexa Fluor^®^ 488- (Thermo Fisher Scientific; A21202) or Alexa Fluor^®^ 594- (Thermo Fisher Scientific; A21207) conjugated secondary antibodies. The cells were washed again with PBST. The coverslips were then placed onto glass slides and mounted with FluoroShield containing DAPI (ImmunoBioScience Corp., Davis, CA, USA).

Images were captured using a Leica DM2500 fluorescence microscope (Leica Microsystems, Wetzlar, Germany) equipped with a Leica DFC450C camera and controlled by Leica Application Suite X (LAS X, version 3.6.0; Leica). Multichannel images were captured at 10× magnification using appropriate excitation/emission settings for each fluorophore. For quantitative comparison, images for each target protein were acquired using identical exposure settings across all experimental groups. Exposure times were individually optimized for each marker to avoid saturation and maximize signal-to-noise ratio (KRT5: 200 ms; KRT1: 100 ms; involucrin: 300 ms; DAPI: 50 ms), with a constant gain of 5.0 for all channels. For each fluorescent channel, data were measured using Image J software (1.54g, National Institutes of Health, USA) and processed with the formula: Corrected total cell fluorescence (CTCF) = Integrated Density (IntDen) − (Area × Mean fluorescence of background readings).

### 4.5. Reverse Transcription–Quantitative PCR (RT-qPCR)

Keratinocytes were lysed in TRIzol reagent (TransGen Biotech, Beijing, China). Total RNA was isolated using the ReliaPrep RNA Miniprep System (Promega, Madison, WI, USA). RNA concentrations were determined using a NanoDrop spectrophotometer (BioTek, Winooski, VT, USA). For each reverse transcription reaction, 3500 ng of total RNA was reverse-transcribed into cDNA using PrimeScript RT Master Mix (Perfect Real Time; TaKaRa, Shiga, Japan). The PCR reaction mixture contained diluted cDNA, 2× PCR Premix (TaKaRa), PrimeScript RT Master Mix (TaKaRa), and primers (sequences listed in Table 2). The reaction mixtures were transferred to 96-well real-time PCR plates (Roche, Basel, Switzerland) and analyzed using the LightCycler 96 System (Roche). Relative gene expression was calculated using the 2^−ΔΔCT^ method, with 18S rRNA as an internal control.

### 4.6. Western Blotting

Keratinocytes were lysed in radioimmunoprecipitation assay buffer (Sigma-Aldrich) containing PhosSTOP™ phosphatase inhibitors (Roche, Basel, Switzerland). Protein concentrations were determined using the BCA protein assay kit. The samples were then mixed with 5× sample loading buffer (GenScript, Piscataway, NJ, USA), denatured, and stored at −80 °C until further analysis.

Proteins (20 μg of total protein per lane) were separated by Bis-Tris polyacrylamide gel electrophoresis (PAGE) using MOPS running buffer (GenScript). The proteins were then transferred onto polyvinylidene difluoride membranes (Merck Millipore, Billerica, MA, USA). The membranes were blocked with 5% skim milk or bovine serum albumin for at least 1 h at room temperature. The membranes were subsequently incubated overnight at 4 °C with primary antibodies diluted in blocking buffer (dilutions listed in Table 3), followed by incubation for 1 h at room temperature with the appropriate horseradish peroxidase-conjugated secondary antibodies (1:2500; Merck Millipore; goat anti-rabbit IgG or goat anti-mouse IgG). The membranes were washed thoroughly with Tris-buffered saline containing Tween-20. Chemiluminescence signals were detected using ECL solution (Claro Sola, Claro Mucho, and Claro SupremoPlus; BioD Co., Gyeonggi-do, Republic of Korea) and visualized using a luminescence imaging system (WSE-6100; ATTO, Tokyo, Japan). Relative protein expression was calculated using GAPDH as a loading control.

### 4.7. Statistical Analysis

All experiments were conducted with at least four independent replicates (n = 4). The control group served as the reference and was normalized to 1.0. Statistical analyses were performed using IBM SPSS Statistics (version 24.0; SPSS Inc., Chicago, IL, USA). The Kruskal–Wallis test was used to assess differences among multiple groups, followed by the Mann–Whitney U test for pairwise comparisons if significant overall differences were detected. The Benjamini–Hochberg procedure was applied to control the false discovery rate (FDR) at 0.05. The raw *p*-values from pairwise comparisons were compared with the corresponding critical values to obtain adjusted *p*-values. Data are presented as mean ± standard deviation, and statistical significance was defined as adjusted *p* < 0.05.

## 5. Conclusions

In summary, our findings indicate that ML281 does not inhibit STK33 in post-burn HTSKs but still alters multiple cellular processes relevant to their pathological phenotype, including proliferation, EMT, differentiation, and apoptosis.

## Figures and Tables

**Table 1:** Demographic characteristics of patients with post-burn hypertrophic scar.

Patients	Location of Specimens (Normal/Scar)	Age (Years)	Sex	Months Post-Burn
1	Abdomen/abdomen	34	Male	13
2	Chest/chest	29	Female	12
3	Thigh/thigh	41	Female	13
4	Flank/abdomen	26	Male	15

**Table 2:** Real-time polymerase chain reaction primer sequences.

Gene	Forward (5′ → 3′)	Reverse (5′ → 3′)
*S18*	GCAGAATCCACGCCAGTACAAG	GCTTGTTGTCCAGACCATTGGC
*STK33*	GCGGTGAAGAAGCAAAGTAGGAG	CGACGCCTATGCTCCAAATGTC
*MYC*	CCTGGTGCTCCATGAGGAGAC	CAGACTCTGACCTTTTGCCAGG
*KRT1*	CAGCATCATTGCTGAGGTCAAGG	CATGTCTGCCAGCAGTGATCTG
*KRT5*	GCTGCCTACATGAACAAGGTGG	ATGGAGAGGACCACTGAGGTGT
*KRT6*	TGAAGAAGGATGTGGATG	ATCATACAAGGCTCTCAG
*KRT10*	CCTGCTTCAGATCGACAATGCC	ATCTCCAGGTCAGCCTTGGTCA
*KRT14*	GCTGAGATCAAAGACTACA	AGAAGGACATTGGCATTG
*KRT16*	CCTACTTCAAGACCATCG	CCTGGCATTGTCAATCTG
*KRT17*	ATCCTGCTGGATGTGAAGACGC	TCCACAATGGTACGCACCTGAC
*IVL*	GGTCCAAGACATTCAACCAGCC	TCTGGACACTGCGGGTGGTTAT
*NOTCH1*	AGCCTCAACGGGTACAAG	TTGACACAAGGGTTGGATTC
*LORICRIN*	GTCTGCGGAGGTGGTTCCTCT	TGCTGGGTCTGGTGGCAGATC
*CASP14*	GGTGGATGTGTTCACGAAGAGG	CCTTCTTGAACCAGCTCTGCTTC
*CDH1*	GCAGACCTTCCTCCCAATAC	TGGGTCGTTGTACTGAATGG
*CDH2*	CCACCTTA AAATCTGCAGGC	CCATGTTACTGTCCAGTTCG
*VIM*	AAAGCGTGGCTGCCAAGAA	ACCTGTCTCCGGTACTCGTTTGA
*SNAI1*	TGCCCTCAAGATGCACATCCGA	GGGACAGGAGAAGGGCTTCTC
*SNAI2*	ATCTGCGGCAAGGCGTTTTCCA	GAGCCCTCAGATTTGACCTGTC
*TWIST1*	GCCAGGTACATCGACTTCCTCT	TCCATCCTCCAGACCGAGAAGG

**Table 3:** Primary antibodies used in Western blotting analysis.

Target	Host	Dilution	Company (Cat. No.)
GAPDH	Rabbit	1:1000	Cell Signaling Technology (Danvers, MAK, USA) (2118S)
GAPDH	Mouse	1:1000	Santa Cruz Technology (Dallas, TX, USA) (sc-47724)
STK33	Mouse	1:500	Santa Cruz Technology (sc-376498)
Keratin1	Rabbit	1:2000	Abcam (Cambridge, UK) (ab93652)
Keratin5	Rabbit	1:2000	Abcam (ab52635)
Keratin6	Mouse	1:2000	Abcam (ab18586)
Keratin10	Mouse	1:500	Santa Cruz Technology (sc-23877)
Keratin14	Rabbit	1:2000	Abcam (ab181595)
Keratin16	Rabbit	1:2000	Abcam (ab76416)
Keratin17	Rabbit	1:1000	Cusabio Technology (Wuhan, Hubei, China) (CSB-442800)
Involucrin	Mouse	1:500	Santa Cruz Technology (sc-21748)
Notch1	Rabbit	1:1000	Abcam (ab52627)
Loricrin	Rabbit	1:1000	Aviva Systems Biology (San Diego, CA, USA) (ARP41738_T100)
Caspase14	Rabbit	1:1000	Abcam (ab174847)
c-Myc	Rabbit	1:1000	Cell Signaling Technology (9402)
PCNA	Mouse	1:1000	Cell Signaling Technology (2586S)
p21	Rabbit	1:1000	Abcam (ab109199)
p27	Rabbit	1:1000	Abcam (ab32034)
E-cadherin	Rabbit	1:1000	Cell Signaling Technology (3195S)
N-cadherin	Mouse	1:1000	Thermo Fisher Scientific (333900)
Vimentin	Mouse	1:3000	Abcam (ab92547)
Snail1	Rabbit	1:1000	Millipore (Burlington, MA, USA) (ABD38)
Slug	Rabbit	1:1000	Cell Signaling Technology (9585S)
Twist1	Rabbit	1:1000	Calbiochem (Burlington, MA, USA) (DR1088)
Cytochrome C	Rabbit	1:1000	Cell Signaling Technology (4272S)
Cleaved caspase3	Rabbit	1:1000	Cell Signaling Technology (9661S)
Bcl-2	Rabbit	1:1000	Abcam (ab196495)
Bad	Rabbit	1:1000	Cell Signaling Technology (9292S)
Bax	Rabbit	1:1000	Abcam (ab199677)
Bid	Rabbit	1:1000	Cell Signaling Technology (8762S)

## Data Availability

For inquiries regarding access to the datasets generated and/or analyzed in this study, please directly correspond with the corresponding author.

## Supplementary Materials

The following supporting information can be downloaded at https://www.mdpi.com/article/10.3390/ijms27188273/s1.
