# Supplementary material for: ML281 Does Not Function as an STK33 Inhibitor but Modulates Proliferation and Differentiation of Keratinocytes Derived from Hypertrophic Scars
Source: Int J Mol Sci. 2026 Sep 17;27(18):8273. doi: 10.3390/ijms27188273 (PMC13607888; doi:10.3390/ijms27188273)
Supplement: Supplementary file 1 [file ijms-27-08273-s001.zip › Supplementary material & method.pdf]

Supplementary Materials and Methods

Cell Migration Assay

HTSKs were seeded at a density of 1 × 10^5 cells into μ-dishes containing 2-well culture-inserts (ibidi GmbH, Planegg, Germany). The culture-inserts were removed after a 2-day culture, and this time point was defined as day 0 that started treatment with 1 or 10 μM ML281. The untreated group was treated with an equal volume of DMSO, and the positive control was treated with 20 μg/mL of TGF-β1 [1]. To exclude the effects of cell proliferation, cells were treated with 5 μg/mL mitomycin C during migration. Images were captured at 0 and 48 h under a light microscope and analyzed using Image J software. The migration of ML281-treated cells was expressed as a percentage of that of untreated control HTSKs, which was normalized to 100%.

Reference

1. Cheng, F.; Shen, Y.; Mohanasundaram, P.; Lindström, M.; Ivaska, J.; Ny, T.; Eriksson, J. E., Vimentin coordinates fibroblast proliferation and keratinocyte differentiation in wound healing via TGF-β-Slug signaling. *Proc Natl Acad Sci U S A* **2016,** 113, (30), E4320-7.
