# Supplementary material for: ML281 Does Not Function as an STK33 Inhibitor but Modulates Proliferation and Differentiation of Keratinocytes Derived from Hypertrophic Scars
Source: Int J Mol Sci. 2026 Sep 17;27(18):8273. doi: 10.3390/ijms27188273 (PMC13607888; doi:10.3390/ijms27188273)
Supplement: Supplementary file 1 [file ijms-27-08273-s001.zip › Supplementary result.pdf]

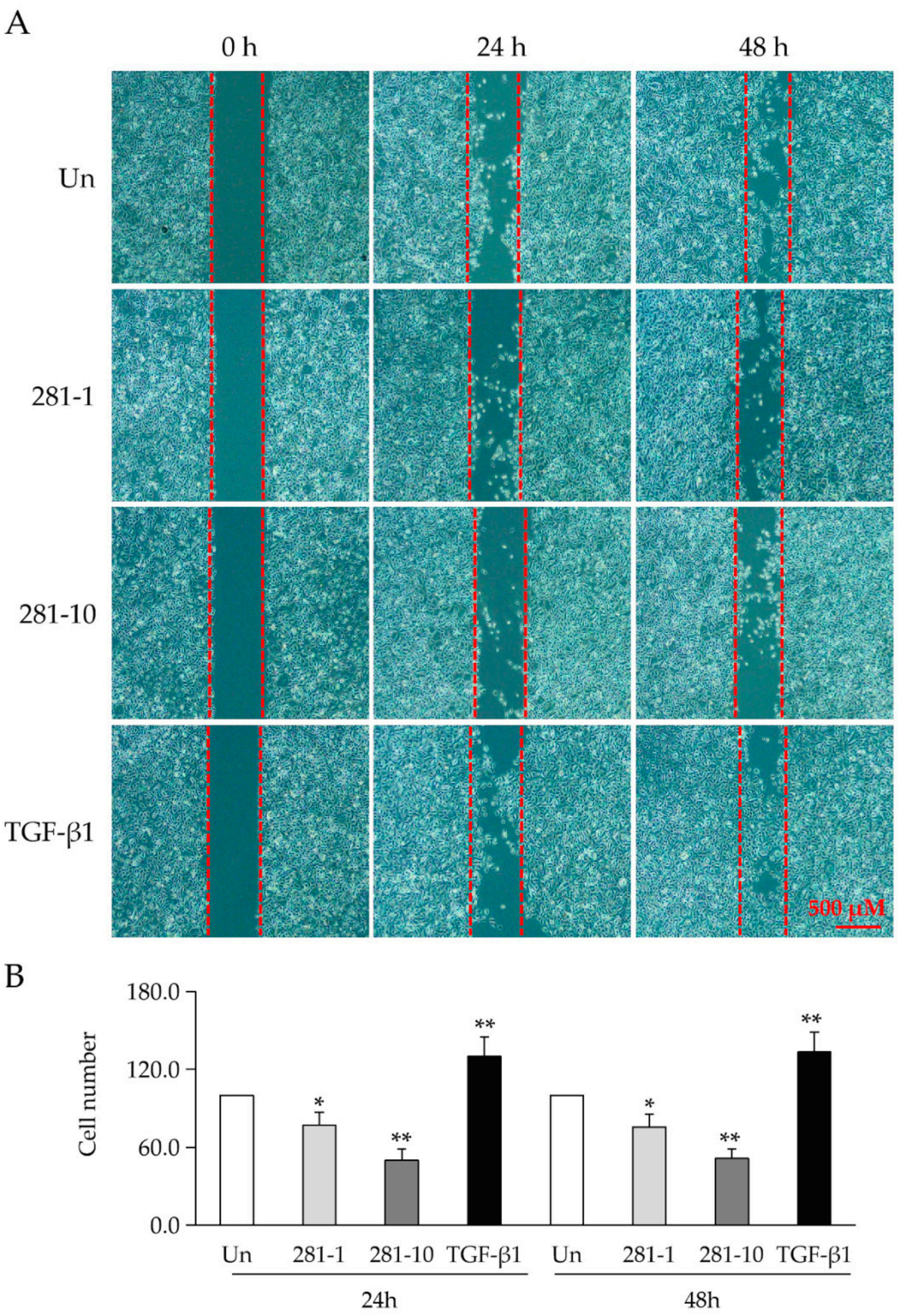

Supplementary Figure S1. ML281 treatment modulated the migration in HTSKs. Treatment with ML281 for 24 and 48 hours significantly reduced cell migration compared with untreated cells, whereas treatment with TGF-β1 significantly promoted cell migration as positive control (A, B). Scale bar = 500 μm. Fold changes were normalized to 100.0 (%) in untreated HTSKs. Each group comprised four biological replicates (n = 4). * $P < 0.05$, ** $P < 0.01$, vs. the untreated group. Un, untreated HTSKs; 1 and 10, ML281 concentrations (μM); TGF-β1, treatment with 20 μg/mL TGF-β1.
